# 3D-printing of dipyridamole/thermoplastic polyurethane materials for bone regeneration

**DOI:** 10.1007/s13346-024-01744-1

**Published:** 2024-11-22

**Authors:** Masoud Adhami, Anushree Ghosh Dastidar, Qonita Kurnia Anjani, Usanee Detamornrat, Quim Tarrés, Marc Delgado-Aguilar, Jonathan G. Acheson, Krishnagoud Manda, Susan A. Clarke, Natalia Moreno-Castellanos, Eneko Larrañeta, Juan Domínguez-Robles

**Affiliations:** 1 School of Pharmacy, Queen’s University Belfast, Lisburn Road 97, Belfast, BT9 7BL United Kingdom; 2 School of Mechanical and Aerospace Engineering, Queen’s University Belfast, Belfast, United Kingdom; 3 Group LEPAMAP-PRODIS, Department of Chemical Engineering, University of Girona, c/M. Aurèlia Campmany 61, Girona, 17003 Spain; 4 Nanotechnology and Integrated Bioengineering Centre (NIBEC), School of Engineering, Ulster University, Belfast, United Kingdom; 5 School of Nursing and Midwifery, Queen’s University Belfast, Belfast, BT9 7BL UK; 6 Department of Basic Sciences, Medicine School, Health Faculty, CICTA, Universidad Industrial de Santander, Cra 27 calle 9, Bucaramanga, 680002 Colombia; 7 Department of Pharmacy and Pharmaceutical Technology, Faculty of Pharmacy, Universidad de Sevilla, Seville, 41012 Spain

**Keywords:** Thermoplastic polyurethane, Fused deposition modelling, 3D printing, Bone regeneration, Dipyridamole, Flexible materials

## Abstract

Tissue engineering combines biology and engineering to develop constructs for repairing or replacing damaged tissues. Over the last few years, this field has seen significant advancements, particularly in bone tissue engineering. 3D printing has revolutionised this field, allowing the fabrication of patient- or defect-specific scaffolds to enhance bone regeneration, thus providing a personalised approach that offers unique control over the shape, size, and structure of 3D-printed constructs. Accordingly, thermoplastic polyurethane (TPU)-based 3D-printed scaffolds loaded with dipyridamole (DIP) were manufactured to evaluate their in vitro osteogenic capacity. The fabricated DIP-loaded TPU-based scaffolds were fully characterised, and their physical and mechanical properties analysed. Moreover, the DIP release profile, the biocompatibility of scaffolds with murine calvaria-derived pre-osteoblastic cells, and the intracellular alkaline phosphatase (ALP) assay to verify osteogenic ability were evaluated. The results suggested that these materials offered an attractive option for preparing bone scaffolds due to their mechanical properties. Indeed, the addition of DIP in concentrations up to 10% did not influence the compression modulus. Moreover, DIP-loaded scaffolds containing the highest DIP cargo (10% w/w) were able to provide sustained drug release for up to 30 days. Furthermore, cell viability, proliferation, and osteogenesis of MC3T3-E1 cells were significantly increased with the highest DIP cargo (10% w/w) compared to the control samples. These promising results suggest that DIP-loaded TPU-based scaffolds may enhance bone regeneration. Combined with the flexibility of 3D printing, this approach has the potential to enable the creation of customized scaffolds tailored to patients’ needs at the point of care in the future.

**Graphical Abstract:**

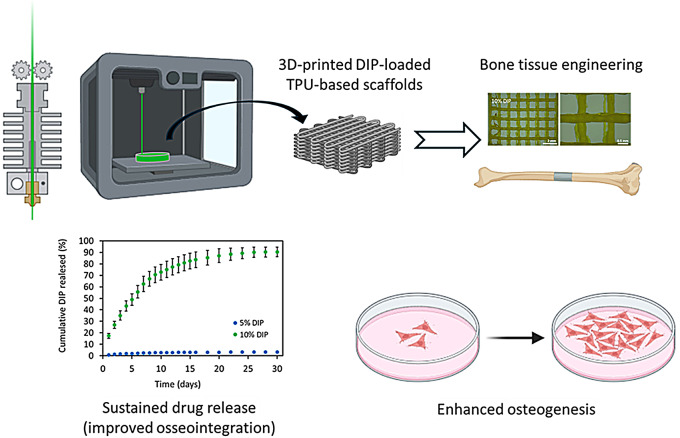

## Introduction

Additive manufacturing, or 3D printing, offers exciting therapeutic approaches in medicine and pharmacy [1–3]. This type of manufacturing technology has been extensively used to prepare a wide range of pharmaceutical formulations, such as oral formulations [4–10], transdermal systems [11, 12] and rectal/vaginal drug delivery systems [13–17]. It has also been used for the development of an extensive range of medical devices, including cardiovascular stents/grafts [18–24], surgical meshes [25–27], subcutaneous implants [28–32], and orthopaedic prostheses [33, 34].

One of the key advantages of additive manufacturing is its versatility, which allows for rapid prototyping and the manufacturing of complex devices in relatively short periods [35]. It offers the possibility of customising and adapting the final object to a patient’s needs. In this way, 3D printing offers a revolution in personalised medicine [35]. 3D printing offers previously unheard-of versatility, with exact control over scaffold’s structure, thus offering tailored solutions to meet the specific architectural, mechanical, and biochemical requirements of the targeted tissue.

Additive manufacturing is an umbrella term that includes a wide range of techniques. The only aspect they have in common is the use of sequential addition of layers of material to produce an object. These techniques include VAT photopolymerization [36], selective laser sintering [37], and fused deposition modelling (FDM) [38], among others. Fused deposition modelling is an exciting technology as it is simple, easy to use, and low cost [39]. Therefore, it can be relatively easy to implement in a clinical scenario. FDM relies on the extrusion of a material through a hot nozzle to add sequential layers of polymer [39]. One area of application for FDM is the development of bone scaffolds and fillers for bone tissue engineering [40].

The development of 3D-printable materials for bone tissue engineering is a highly attractive research area [41–43]. These materials range from biopolymers such as alginate, collagen, or chitosan to ceramic materials such as hydroxyapatite [44, 45]. However, synthetic polymers such as poly(lactic acid), polylactic-co-glycolic acid, silicone, or thermoplastic polyurethane (TPU) have also been extensively used in bone tissue engineering [43, 46–49]. 3D printing offers the possibility of combining any of these materials with drugs to achieve better tissue regeneration, prevent infection/inflammation, or enhance the integration of the implant with the biological tissue [38, 50]. Dipyridamole (DIP) has been reported to be a potential candidate to enhance bone regeneration. DIP is an antiplatelet drug that acts as an agonist of adenosine A2A receptors on osteogenic progenitorsand, therefore, promotes their growth and differentiation [51]. Moreover, DIP has demonstrated promising results in filling bone defects and enhancing bone regeneration with comparable efficacy to BMP-2, one of the most potent bone morphogenetic proteins (BMPs) used for this purpose [52, 53]. Notably, the use of DIP achieved these effects without the concerning side effects associated with BMPs, such as osteolysis, ectopic bone formation, and craniosynostosis, as frequently reported in the literature [53–56].

To date, multiple studies have been conducted in vivo on 3D-printed materials loaded with DIP for bone regeneration [53, 57]. However, most of them used extrusion-based 3D printing techniques that are not simple to transfer to a clinical setup and require the use of expensive 3D printing systems. Moreover, bone conduction scaffolds are fundamental to bone healing, along with essential factors like osteoblast-related cells, and a stable mechanical environment [48]. In this work, we describe the use of FDM for the preparation of TPU-based materials loaded with DIP, a promising osteoinductive agent for bone regeneration strategies. Prior to the 3D printing process, drug-loaded TPU filaments were prepared. Subsequently, different samples were prepared using FDM, and the resulting samples were characterized using various techniques, such as such as scanning electron microscopy (SEM), Raman and Fourier-transform infrared (FTIR) spectroscopy, dynamic mechanical analysis (DMA) and differential scanning calorimetry (DSC), as well as X-ray microcomputed tomography to assess their physicochemical and mechanical properties. Additionally, the release kinetics of DIP over 30 days and in vitro cytocompatibility were evaluated. Finally, the ability of these materials to stimulate osteoblast proliferation was evaluated.

## Materials and methods

### Materials

Elastollan^®^ thermoplastic polyurethane (TPU) with a shore hardness of 80 A, indicating moderate flexibility and resistance to deformation, was kindly provided by DistruPol Ltd (Dublin, Ireland). Dipyridamole (DIP) was acquired from Tokyo chemical Industry (Oxford, UK). Phosphate buffer solution (PBS) was purchased from VWR Chemicals (Ohio, USA). Fetal calf serum (FCS; ThermoFisher Scientific, USA), alpha-minimum essential medium (α-MEM), L-glutamine, penicillin/streptomycin, b-glycerophosphate (BGP) and ascorbic-acid-2-phosphate were purchase from Sigma (St. Louis, MO, USA). All materials and reagents were used as received.

### TPU composite and filament preparation

The process of preparing the TPU composite involved incorporating DIP into the TPU matrix before extruding the drug-loaded filaments. This ensured even distribution of DIP within the TPU matrix, allowing the successful extrusion of DIP-loaded filaments. DIP contents of 5% and 10% were achieved while maintaining homogeneity, using a preheated plastograph Brabender (Brabender GmbH & Co KG, Brabender Plastograph EC, Germany), following our previously published methodology [38]. More detailed information about all the steps can be found in this work. For this purpose, TPU was dried at 80 °C for 48 h in an oven before processing. Finally, the material was milled into particles of approximately 5 mm using an IKA blade mill (MF 10.2 Impact grinding, France). These particles were kept in a container at 80 °C until further processing. TPU-based filaments of 2.85 mm diameter were manufactured using a 3devo model NEXT 1.0 filament extruder (3devo, Utrecht, The Netherlands). Temperatures for blank TPU-based filaments were set between 170 °C and 180 °C, and between 125 °C and 130 °C for DIP-loaded filaments (5% and 10% w/w of DIP). The extruder speed remained constant at 4.5–5.0 rpm regardless of the composite material composition.

### 3D-printed pieces design and manufacture

Various objects, including discs (13 mm diameter), meshes (10 mm × 10 mm), and rectangular-shaped blocks (30 mm × 8 mm × 4 mm), were designed using computer-aided design (CAD) software and 3D printed using an Ultimaker 3 (Ultimaker B.V., Geldermalsen, Netherlands) equipped with a 0.4 mm nozzle. The 3D printing process was performed using the Cura^®^ 3.0 software. For this purpose, a print temperature of 200 °C and a print speed of 12 mm/s were used. The layer height was set at 0.2 mm, while the infill density was set at 100% for all the filaments regardless of the DIP-loaded amount.

### Microscopy

The uniformity of the 3D-printed objects (meshes and rectangular-shaped blocks) was assessed using a Leica EZ4 D digital microscope (Leica, Wetzlar, Germany). The surface morphology of the 3D-printed objects (meshes and rectangular-shaped blocks) was also evaluated by using scanning electronic microscopy (SEM) (Hitachi TM3030; Tokyo, Japan). EasySAM acoustic microscope (Kibero, Saarbrücken, Germany) equipped with an LD 200 acoustic lens (200 MHz) was used to qualitatively evaluate the topography of 3D-printed samples.

### Dynamic mechanical analysis and differential scanning calorimetry

Dynamic Mechanical Analysis (DMA) measures the mechanical properties of materials as a function of temperature, time, and frequency. This technique helps to assess the viscoelastic behavior of materials, providing insights into their stiffness, damping characteristics, and transitions between solid and liquid states. DMA measurements were carried out according to standard ASTM D4065 and D7028-07. The measurements were taken using a Triton Technology Tritec 2000 DMA with a single cantilever set-up used. The 3D-printed samples were run in the temperature range of -60 °C to 100 °C, using a frequency of 1 Hz and deformation of 0.02 mm. To cool the sample down to -60 °C, liquid nitrogen was used, and the samples were heated to 100 °C using a heating rate of 2 °C/min. The dimensions of the 3D-printed samples were 10 mm x 10 mm x 4 mm.

Differential Scanning Calorimetry (DSC) is a thermal analysis technique that measures the heat flow associated with phase transitions of materials as a function of temperature. It is commonly used to analyze thermal properties of such as melting points or glass transition temperatures. DSC was used to examine how the pure DIP in the 3D printed scaffolds responded to temperature fluctuations. A DSC 214 Polyma (NETZSCH, Munchin, Germany) was used to assess the thermal behaviour of the printlets. After precisely measuring a small amount (between 5 mg and 10 mg), the aluminium pans were placed and sealed to begin the DSC analyses of the various samples. The reference was an empty, sealed aluminium pan. Utilising a heating rate of 10 °C/min and a nitrogen flow rate of 50 mL/min, the system was run at temperatures between − 60 and 300 °C.

### Attenuated total reflection fourier transform infra-red (FTIR) Spectroscopy

Pure DIP and two different loaded DIP scaffolds were analysed using Fourier transform infrared spectroscopy (FTIR) using the Nicolet™ iS50 ATR instrument (Thermo Scientific™, USA)). At room temperature, the IR spectra were collected and scanned in the range of 4000 to 600 cm^− 1^. Spectra were acquired by averaging 64 scans, with resolution kept constant at 4.0 cm^− 1^ throughout the investigation.

### Raman spectroscopy/microscopy

DIP-loaded blocks in this study were characterised using a TA RM5 Raman Microscope (Edinburgh Instruments, Edinburgh, UK) with a 785 nm laser. A 300 mm pinhole, 70 mm slit, and full laser intensity (100%) were applied. The field of view captured with a 10x objective was mapped using a 15 × 15 point grid to evaluate DIP distribution. At each grid point, a single Raman spectrum was captured with a 2.5-second exposure time and three accumulations. Spectral background was removed using Ramacle software (Edinburgh Instruments, Edinburgh, UK). To visualise DIP distribution, the intensity of DIP’s characteristic peak at approximately 1375 cm⁻¹ was plotted across individual grid points.

### Mechanical properties

Compression Modulus of the 3D-printed materials were evaluated with the assistance of a TA.XTplus texture analyser (Stable Micro Systems, Surrey, UK). For this purpose, blocks with a dimension of 10 mm on each side were printed. The TA.XTplus texture analyser was configured to operate in compression mode, and the blocks were compressed at a constant pace of 2 mm/sec between the probe and an aluminium block for a distance of 5 mm. The compression modulus was calculated using the slope of the linear part of the stress/strain graphs.

### Optical coherence tomography and microcomputed tomography

Optical coherence tomography (OCT) was used to analyse the surface and outer layers of 3D-printed samples. For this purpose, VivoSight™ Topical Multi-Beam OCT Handheld Probe (Michelson Diagnostics Ltd, Kent, UK) was used. A surface of 6 × 6 mm was analysed recording 250 frames (distance between frames: 0.024 mm). OCT was used to qualitatively evaluate the topography of 3D-printed samples.

X-ray microcomputed tomography (µCT) was conducted using a Bruker Skyscan 1275 (Bruker, Kontich, Belgium) equipped with a Hamamatsu L11871 source. The analysis was performed at a voltage of 80 kV and an amperage of 87 µA. The Bruker CTvol software was utilised to carry out the process of volumetric reconstruction. To reduce the speckle surrounding the samples, attenuation thresholding was done manually. µCT was used to qualitatively assess the morphology of the sample’s inner layers.

### In vitro release study

An in vitro release study was carried out to determine the amount of DIP eluted from 3D-printed meshes. The meshes were weighed, and their weight ranged from 33.5 mg to 38.2 mg for the formulation containing 5% w/w of DIP, and from 28.8 mg to 36.2 mg for the formulation containing 10% w/w of DIP. For this purpose, meshes containing DIP at different concentrations (5% w/w and 10% w/w) were immersed in different PBS volumes (pH 7.4) to maintain the sink condition. The PBS volumes varied from 20 mL to 2 mL for the meshes containing 5% w/w of DIP and from 500 mL to 10 mL for the meshes containing 10% w/w of DIP, with the volume being progressively reduced. The jars containing meshes were shaken at 40 rpm at 37 °C for the 30 days. Samples of the release medium were taken at predetermined time and replaced with fresh medium. All the reported time points are a mean of four measurements over 30 days. The concentration of DIP from each samples was analysed using fluorescence spectroscopy (FLUOstar Omega Microplate Reader, *BMG* LABTECH, Ortenberg, Germany) with the excitation and emission wavelength were 280 nm and 460 nm, respectively.

### In vitro biocompatibility studies

MC3T3-E1 cells, which are a murine calvaria-derived pre-osteoblastic cell line [58], were used as a cell model in this work. MC3T3-E1 were incubated at 37 °C and 5% CO_2_ in alpha-minimum essential medium (α-MEM; Sigma, St. Louis, MO, USA), supplemented with 10% fetal calf serum (FCS; ThermoFisher Scientific, Waltham, MA, USA), 1% (w/v) L-glutamine (Sigma, St. Louis, MO, USA), and 1% (w/v) penicillin/streptomycin (P/S; Sigma, St. Louis, MO, USA), referred to as α-MEM+, which was changed every other day. 10 mM b-glycerophosphate (BGP; Sigma, St. Louis, MO, USA) and 50 µg/mL ascorbic-acid-2-phosphate (Sigma, St. Louis, MO, USA) were added to the complete medium to induce osteoblast differentiation (Osteogenic Medium: OM).

Disc shaped 3D printed samples (10 mm x 4 mm) were sterilised with UV-C irradiation for 45 min. Subsequently, the cells were seeded onto samples with a density of 5000 cells/cm^2^ in a 12-well plate and incubated for 7 days. Following this, the samples were further evaluated using a cell viability test, a metabolic activity test, a lactate dehydrogenase (LDH) assay, and a cell proliferation test.

Cell viability in response to samples of (Control cells/ TPU Blank/ TPU DIP 5%/ TPU DIP 10%) were measured using MTT (3-[4,5-dimethylthiazol-2-yl]-2, 5 diphenyltetrazolium) assay as was described before [59]. Briefly, cells were seeded at a density of 5000 cells/cm2 and the cell activity was evaluated after 7 days of culture, the cell seeded samples were washed with PBS. Then, MTT solution (0.5 mg/mL) was added to each well followed by incubated for 5 h at 37 °C with 5% CO_2_. The supernatant was removed gently, followed by the addition of dimethyl sulfoxide (DMSO). Cells hidden on the plate without any treatment were used as control cells. The absorbance was read at 570 nm on a Synergy H1 microplate reader (Agilent Technologies, St. Clara, USA) (Biotek, Winooski, VT, USA).

Proliferation was measured using the DNA content assay as was described before [60] on Control cells, TPU Blank, TPU DIP 5% and, TPU DIP 10%. The amount of DNA in the cells attached to the biomaterials was determined using Quant-iT™ PicoGreen^®^ dsDNA Reagent and Kits (Molecular Probes, Life Technologies Corp.) according to the manufacturer’s instructions. The samples were rinsed with PBS three times and submerged in 1 mL of lysis buffer containing 10 mM Tris (pH 8), 1 mM EDTA, and 0.2% (v/v) Triton X-100. To release the DNA, the samples were vortexed for 10 s every 5 min for a total of 30 min and were kept on ice throughout the entire process. The samples were thawed on ice, and homogenized 10–15, after, the sample was mixed with 100 µL of DNA-binding fluorescent dye solution. The fluorescence intensity was measured at an excitation wavelength of 480 nm and an emission wavelength of 520 nm. A standard curve was constructed using Lambda DNA order to quantify the amount of DNA that is being assayed.

The alkaline phosphatase (ALP) assay was performed with MC3T3-E1 osteoblast cells cultured on (Control cells/ TPU Blank/ TPU DIP 5%/TPU DIP 10%). The assay was conducted using a Sensolyte^®^ pNPP alkaline phosphatase assay kit (AnaSpec, USA). Cells were detached from scaffolds in a 500 µL lysis buffer, provided in the kit, for 10 min. Lysates were centrifuged at 2500× g at 4 °C for 10 min. Supernatant was collected for the ALP assay using p-nitrophenyl phosphate (pNPP) as substrate. Each specimen was incubated with the addition of 100 µL of a pNPP solution at 37 °C for 30 min. The production of p-nitrophenol in the presence of ALP was measured by monitoring light absorbance by solution at 405 nm, as manufacturer’s instruction. The results were normalized to the amount of ALP provided in the kit as a standard.

### Statistical analysis

Data were presented as the average ± standard deviation of at least three replicates. Statistical analysis was carried out with one-way ANOVA followed by a Tukey post-hoc test. In all cases, *p* < 0.05 was accepted as statistically significant difference. For the release study, an unpaired t-test was used to compare the release values of DIP from both 3D-printed scaffolds formulations (5% w/w and 10% w/w).

## Results and discussion

TPU has been extensively used for bone and cartilage tissue engineering [61–63]. TPU is a family of polymers offering a wide range of properties depending on their chemical composition. The TPU selected for this work is a non-degradable material. The use of non-degradable TPU has been reported previously in bone engineering and other orthopedic applications [62]. These materials offers an attractive option to prepare bone scaffolds due to its mechanical properties, good biocompatibility and because it is easy to process [64]. Accordingly, TPU can be used for 3D-printing applications. This material is commonly used for extrusion-based 3D-printing such as FDM [20, 65, 66]. As mentioned earlier, 3D-printing allows the possibility of preparing prostheses adapted to patient’s needs. An attractive application is the development of joint prosthesis. Flexible materials such as TPU or silicone have been used for the development of small joint prostheses for arthroplasty [67]. There are commercial silicone implants such as NeuFlex^®^ or Swanson^®^ in different sizes [68]. However, they are not adapted to the anatomy of the patient. Also, the addition of DIP in the material enhances osseointegration with bone following implantation, improving the prosthesis’ stability and function. It is important to mention that the TPU used in this study is non-biodegradable and therefore it is critical to improve osseointegration of the material. This is one of the potential applications of the materials developed in this work. However, there are other potential applications. Another potential application will be the production of customised scaffolds can be produced to treat critical-size defects, tailored precisely to the defect’s shape and size.

### Physicochemical characterization of 3D-printed samples

**Fig. 1:**
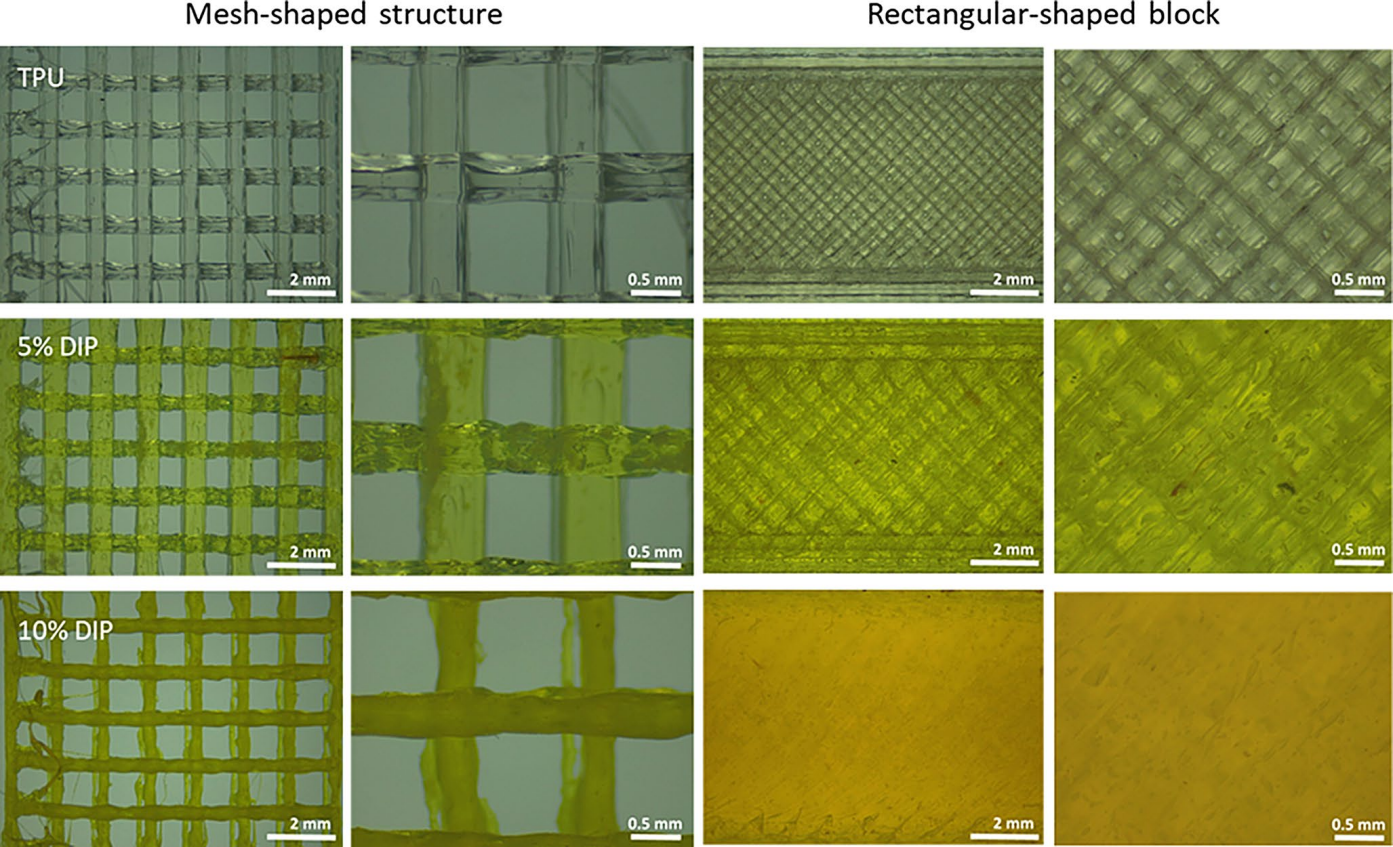
Optical microscopy images of different 3D-printed structures printed using different TPU/DIP combinations

The initial stage involved a pre-extrusion process to incorporate 5% w/w and 10% w/w of the drug into the polymeric matrix while ensuring the homogeneity and consistency in the resulting materials, achieved using a plastograph Brabender. Subsequently, hot-melt extrusion (HME) was employed to manufacture the TPU and DIP-loaded TPU-based filaments, which were used to prepare the different 3D-printed materials, including discs, meshes, and rectangular-shaped blocks, by FDM. The yellow tone of the 3D-printed mesh- and rectangular-shaped structures, observed through a light microscope (Leica, Wetzlar, Germany), intensified consistently with the increase in DIP content (from 5 to 10% w/w), confirming higher DIP concentrations within the 3D-printed structures (Fig. 1). Indeed, 3D-printed samples with 10% DIP concentration demonstrated greater opacity compared to samples comprising solely TPU or 5% DIP (Fig. 1). Furthermore, these 3D-printed structures were quite homogenous, without any visible DIP clumps or aggregates. Thus, this is suggesting that the drug was successfully combined with the TPU matrix. Moreover, it can be inferred that, a complete mixing process has been performed, which is facilitated by the high temperature and intense shear forces generated by the rotating screw actions within both the pre-extrusion and single screw extruder [69]. Additionally, these results suggest that FDM was successfully used to prepare objects with different infill densities using TPU/DIP combinations.

Interestingly, samples containing 10% DIP showed thinner strands of TPU-based material when preparing mesh-shape structures. This indicates that the viscosity of this TPU/DIP composition when melted will be different than the viscosity of pristine TPU. This has a clear influence on the printing process as can be seen in Fig. 1.

**Fig. 2:**
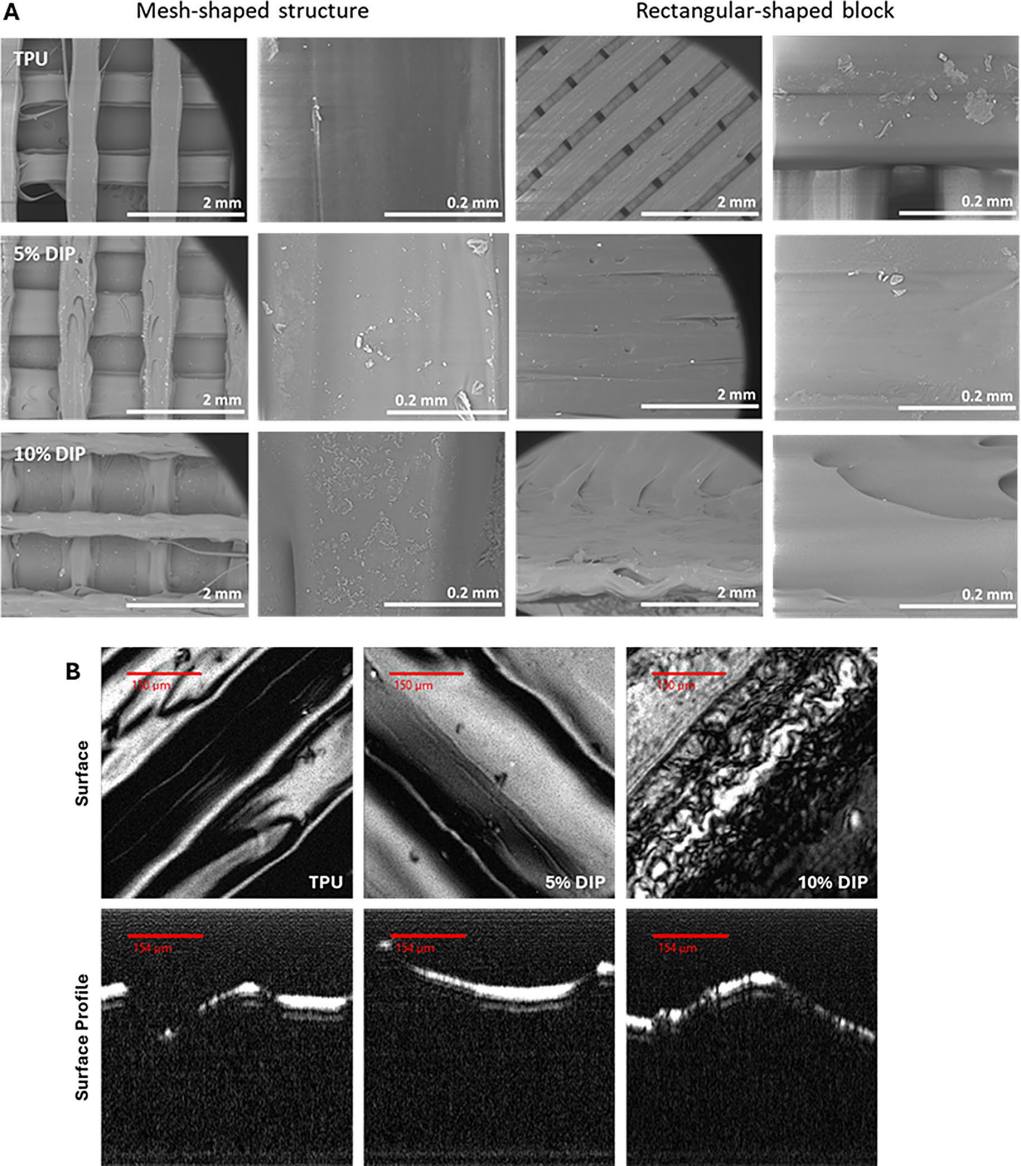
SEM micrographs of different 3D-printed structures printed using different TPU/DIP combinations (**A**). Acoustic microscope images of the surface and Surface Profile of 3D-printed blocks prepared using different TPU/DIP combinations (**B**). Scale bars for acoustic microscopy images: 150 μm for top panels and 154 μm for bottom panels

Surface characterization of the 3D-printed mesh and rectangular-shaped structures was conducted *via* SEM analysis. The SEM images (Fig. 2A) revealed that 3D-printed samples fabricated without DIP and those with 5% DIP exhibited a smoother surface in comparison with 3D-printed samples containing 10% w/w DIP, which displayed increased surface roughness. Thus, the surface morphology appeared to be influenced by the drug concentration, aligning with previous literature findings [38, 70, 71].

**Fig. 3:**
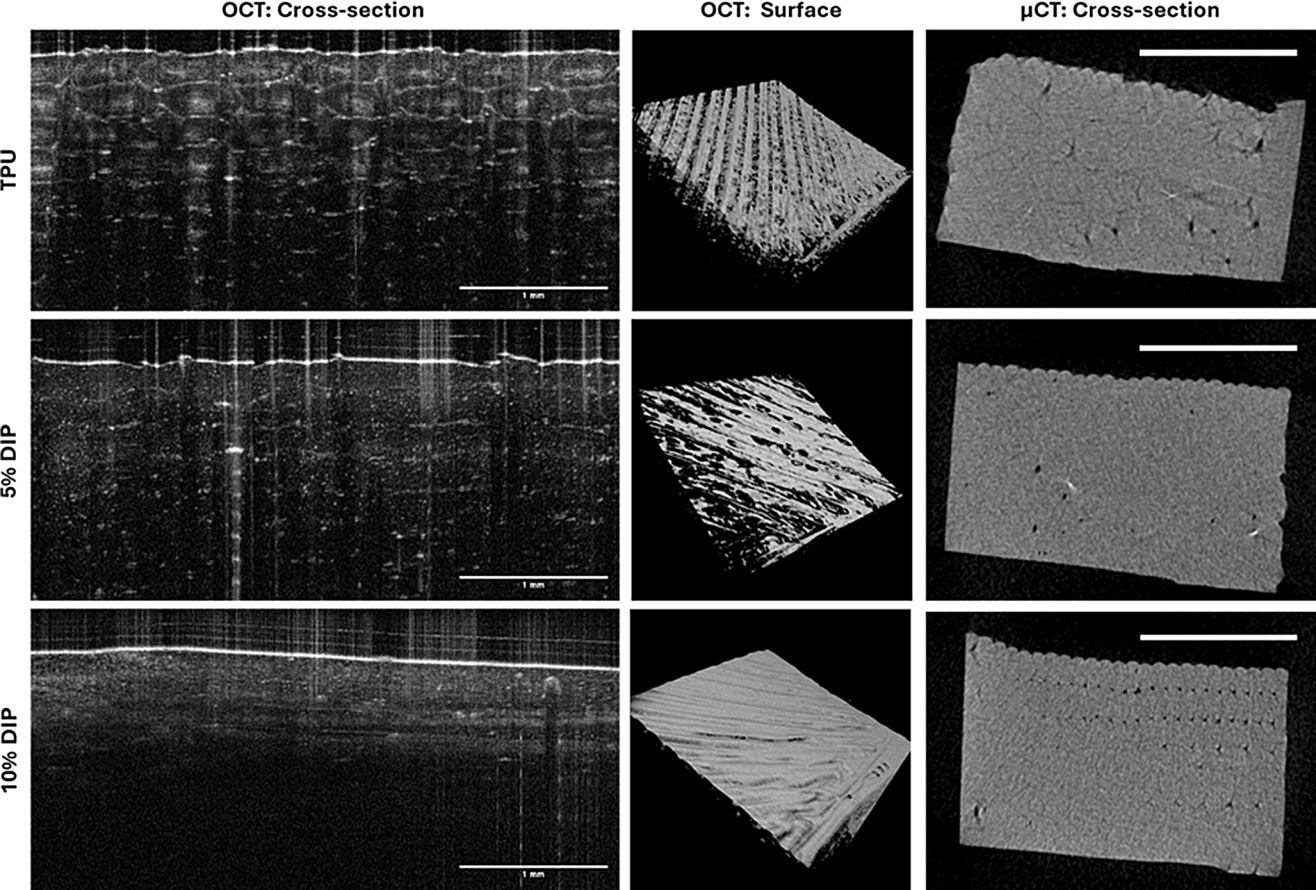
OCT and µCT images of 3D-printed structures containing different TPU/DIP combinations. Scale bars: OCT-Cross-section: 1 mm; OCT-Surface: 6 × 6 mm surface; µCT-Cross-section: 5 mm

Additionally, acoustic microscopy was used to evaluate surface morphology and topography of 3D-printed samples (Fig. 2B). Acoustic microscopy provides not only an image of the surface of the device but also about the surface profile [72]. The results obtained are in line with the results described previously, the inclusion of DIP increase surface roughness. The effect is especially noticeable for samples containing 10% DIP. The surface profile is noticeably rougher than the surface profile of the pristine TPU or samples containing 5% DIP. OCT was used to evaluate the surface profile at a larger scale (Fig. 3). This is a useful technique to characterise medical devices such as vaginal rings/inserts [15, 73, 74], microneedles [75, 76] or subcutaneous implants [31, 77, 78]. The cross-sectional OCT images showed the layered structure characteristic of 3D-printed materials for TPU samples. The layers can be seen too for 5% but it is obvious that there was a better integration between the layers. Finally, when the drug loading increased up to 10% the layered structure cannot be distinguished in the OCT cross-sectional images. Additionally, as drug content increased the laser penetration was reduced as can be seen in the definition of the images at deeper layers. Similarly to acoustic microscopy, OCT can be used to evaluate the surface morphology. The 3D-printing patter can be easily distinguished in the TPU samples. However, the TPU strands showed a better integration between them when TPU was added. On the other hand, µCT was used to evaluate internal structure of 3D-printed samples (Fig. 3). In this case X-rays penetrate through the material giving information about the internal structure. This technique is not limited to the top layers like with OCT that relies on laser penetration into the material. The results suggest that the obtained structure presented homogeneous internal structure and all the layers were fused together as no obvious bubbles or voids were detected.

**Fig. 4:**
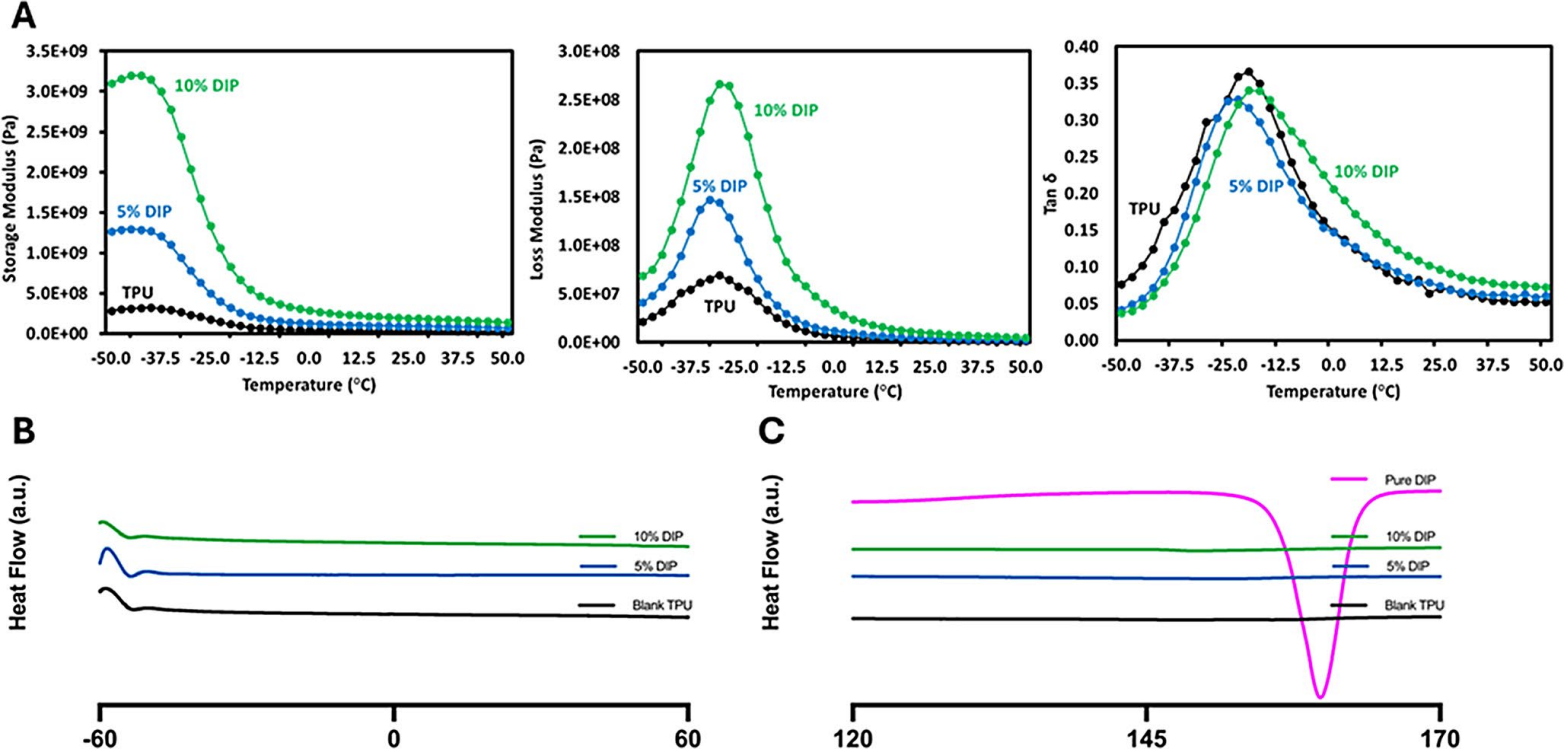
DMA curves obtained for different TPU/DIP 3D-printed samples (**A**). DSC curves for TPU/DIP 3D-printed samples: TPU T_g_ region (**B**) and DIP melting point region (**C**)

To evaluate DIP and TPU interactions within the 3D-printed samples DMA (Fig. 4A) and DSC (Fig. 4B-C) were performed. Figure 4A shows the results of the DMA analysis including comparative storage modulus - temperature and Tan δ -temperature curves for the neat TPU and TPU/DIP composites. The storage modulus of the TPU samples in the − 50/+50 °C temperature range increased with the DIP concentration in both the glassy and rubbery areas. Interestingly, the storage modulus values in the glassy areas of the polymer increased with DIP concentration. The storage modulus determines the elastic energy that materials store during deformation and provides details on the stiffness of polymers. These results indicate that the drug interacts with the polymer reinforcing the resulting material. These results are consistent with previously reported results for poly(caprolactone)-DIP combinations [21]. All samples showed a distinct and well-defined T_g_ in the Tan δ -temperature curves (Fig. 4A). It is important to note that there is no obvious T_g_ change when DIP was included in the mixture. Tan δ is a measure of energy dissipation from the material that describes the ratio of loss modulus to storage modulus. It is interesting to note that TPU had a higher Tan δ at low temperatures that dropped rapidly as the temperature increased. However, both the composites of TPU-DIP possessed lower Tan δ values than pure TPU.

DSC analysis (Fig. 4B-C) was performed focusing on two different areas: the glass transition temperature zone for TPU (Fig. 4B) and the DIP melting point (Fig. 4C). DSC did not show an obvious T_g_ for the sample in the region between − 50 and 0 °C. DMA provide a better indication of T_g_ as mechanical changes are more dramatic than changes in heat capacity [79]. Alternatively, TPU/DIP samples did not show the characteristic melting point for DIP (Fig. 4C). This indicates that the drug is in amorphous state when combined with TPU indicating that interactions took place between the drug and the polymer. These results are consistent with the changes in storage modulus obtained for DIP/TPU samples when compared with pristine TPU samples. Moreover, DIP has shown similar behavior when combined with other polymers with drug loadings ranging between 5 and 20% [21].

**Fig. 5:**
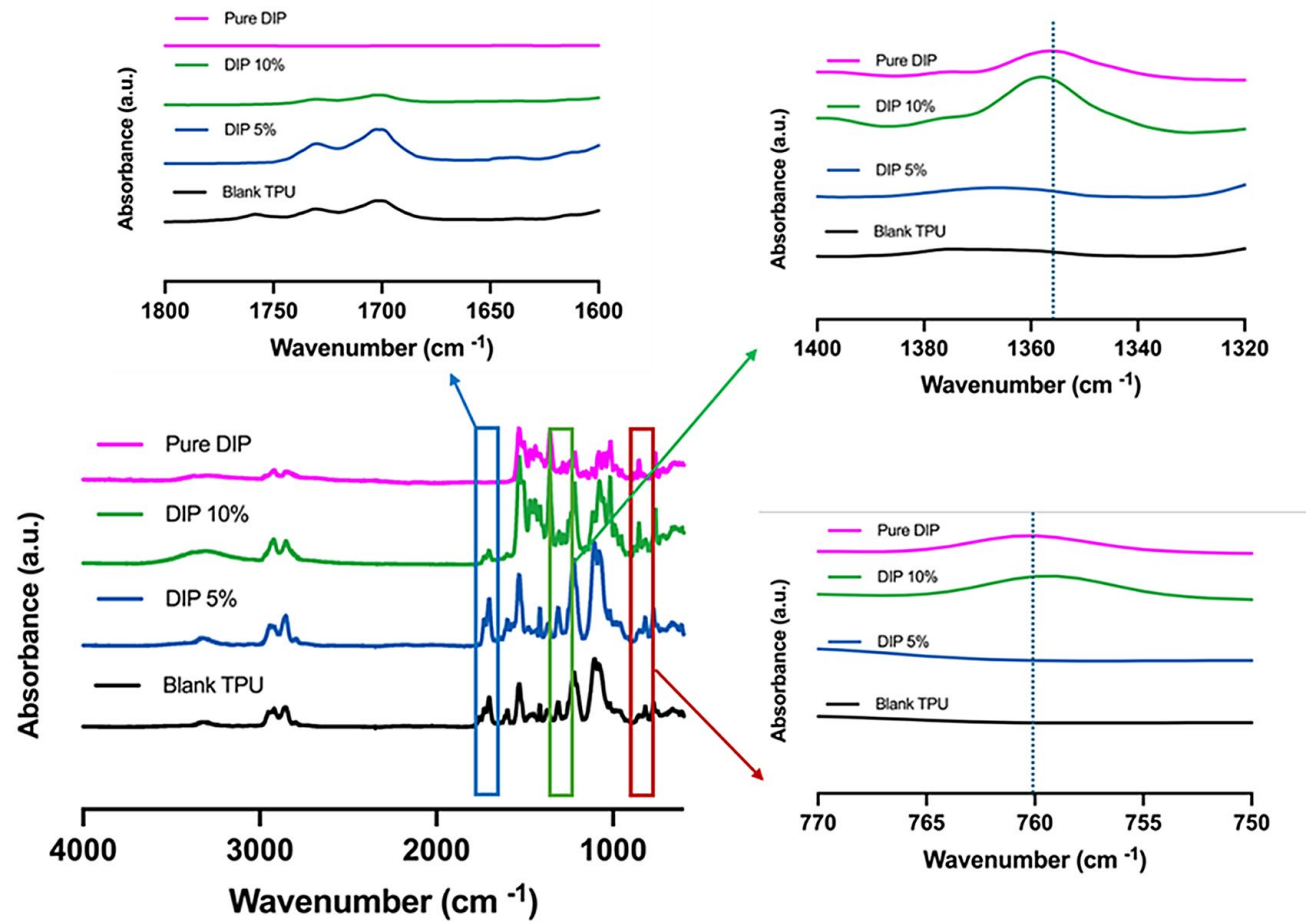
FTIR spectra of pure DIP, pure TPU and 3D-printed samples containing different DIP/TPU combinations

**Fig. 6:**
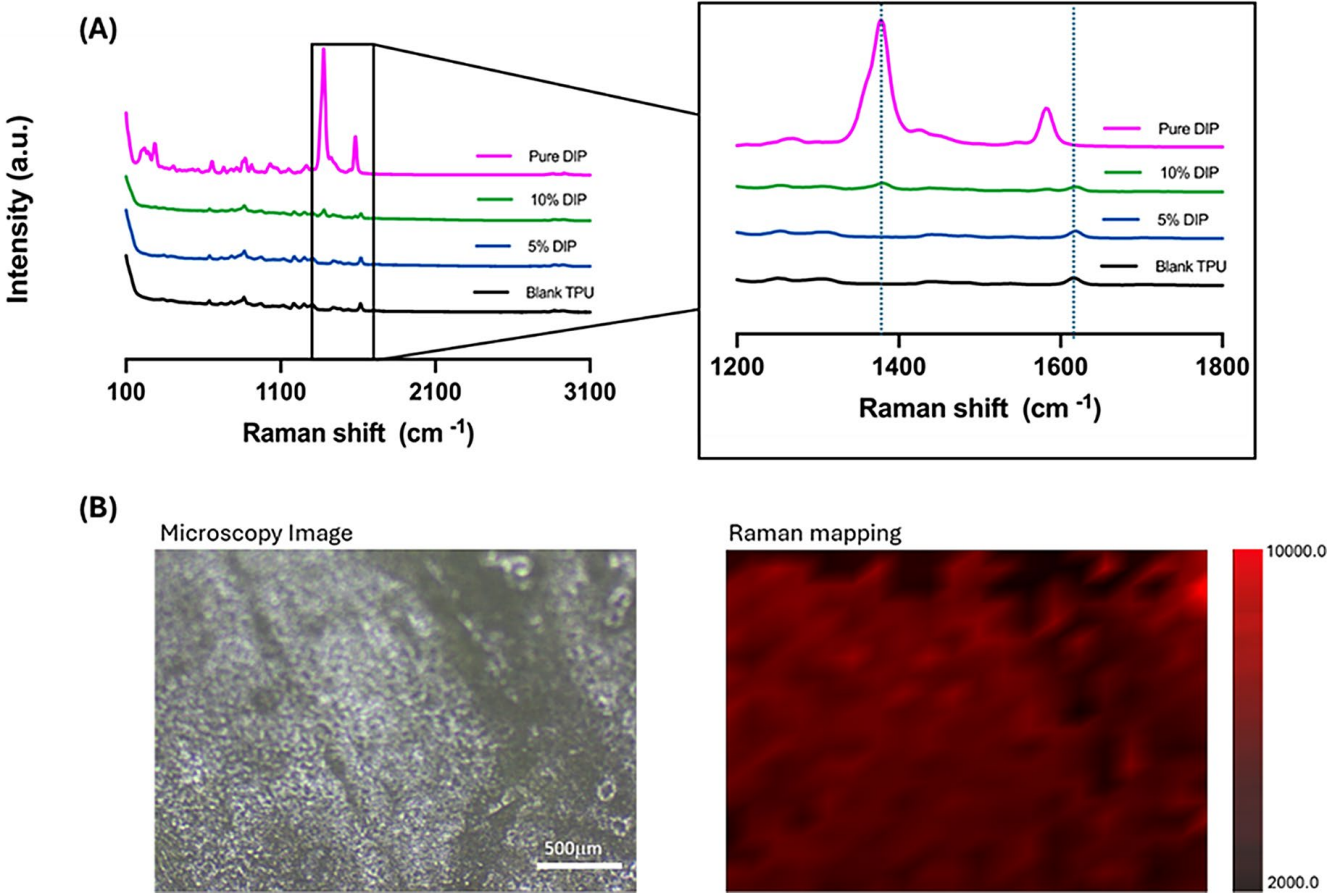
Raman spectra of pure DIP, pure TPU and 3D-printed samples containing different DIP/TPU combinations (**A**). Microscopy image of a 3D-printed sample containing 10% DIP (left panel) and Raman map of the sample surface displaying the intensity of the DIP peak at 1350–1400 cm^− 1^ (right panel) (**B**)

FTIR spectra (Fig. 5) of the samples showed that no chemical reactions took place between TPU and DIP as no new peaks were observed. Alternatively, samples containing 5% DIP displayed similar bands to pristine TPU. The DIP loading was too low to be able to be seen in the samples. Interestingly, samples containing 10% DIP showed obvious differences in the FTIR spectrum. The peaks assigned to DIP were noticeable in the sample as well as some characteristic peaks assigned to TPU such as the C = O bond (ca. 1700 cm^− 1^). The intensity of the carbonyl peak decreases for samples containing 10% DIP. Also, these samples showed shifts in DIP characteristic peaks attributed to the C-N bonds (ca. 1350 cm^− 1^) and C-C-O bonds (ca. 760 cm^− 1^) showed shifts. These results suggest the formation of non-covalent interactions between TPU and DIP. These results are in line with previously reported combinations of DIP with other polymers such as poly(caprolactone) or poly(vinyl pyrrolidone) [21, 80]. Interestingly, Raman spectra (Fig. 6) of the samples showed similar results. The peak attributed to DIP between 1350 and 1400 cm^− 1^ (C-N bonds) was observed in the Raman spectra of samples containing 10% DIP. This peak showed a clear shift in the wavenumber confirming interactions with TPU. Alternatively, the carbonyl peak for TPU was observed in all samples (ca. 1600 cm^− 1^). This peak showed a clear shift indicating that TPU C = O took place in the interactions between the drug and the polymer. These results confirm the interactions reported previously between DIP and TPU for DMA and DSC results. Finally, Raman mapping (Fig. 6) was used in 3D-printed samples containing 10% DIP to confirm what was observed in the microscopy evaluation of the samples: DIP was evenly distributed through the sample (Fig. 6B).

**Fig. 7:**
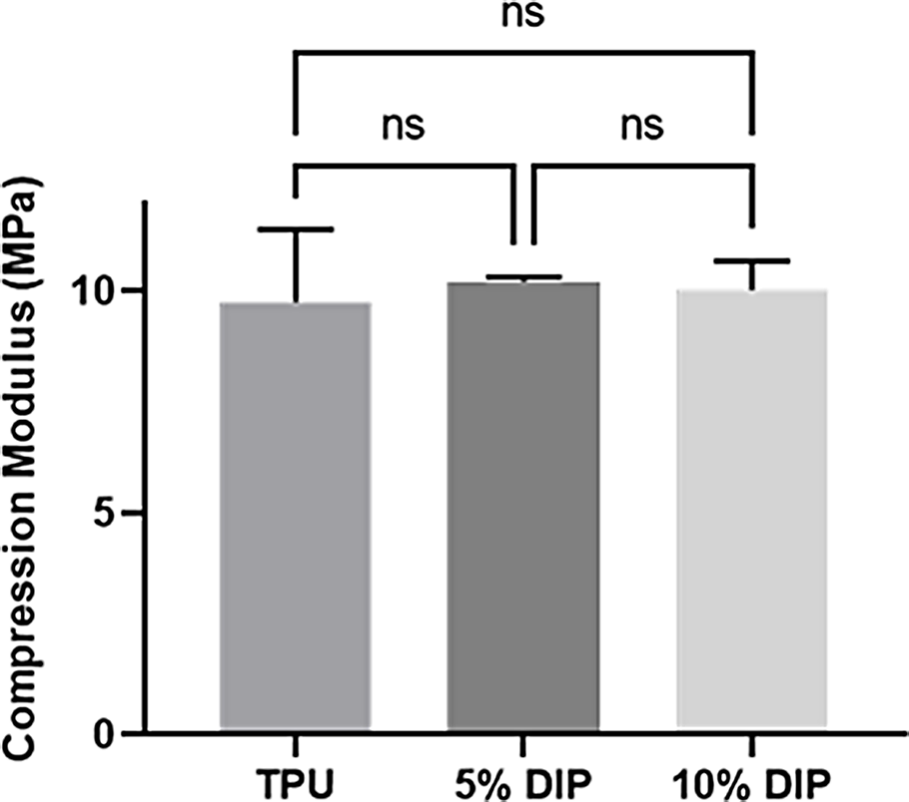
Compression modulus of 3D-printed samples containing different TPU/DIP combinations

Figure 7 shows the results obtained for mechanical testing of the resulting 3D printed materials. The results indicated that the compression modulus in all the samples was around 10 MPa for all TPU/DIP composition. Accordingly, it can be established that the addition of DIP in concentrations up to 10% did not influence compression modulus. The literature presents multiple examples of harder materials or combinations of TPU with fillers to achieve stiffer materials. However, it is important to note that there are softer materials used in 3D-printing applications for bone tissue engineering such as hydrogels (alginates, collagen or gelatin among many others) [81] or commercial bioinks such as OsteoInk^®^ [82].

### Release study

**Fig. 8:**
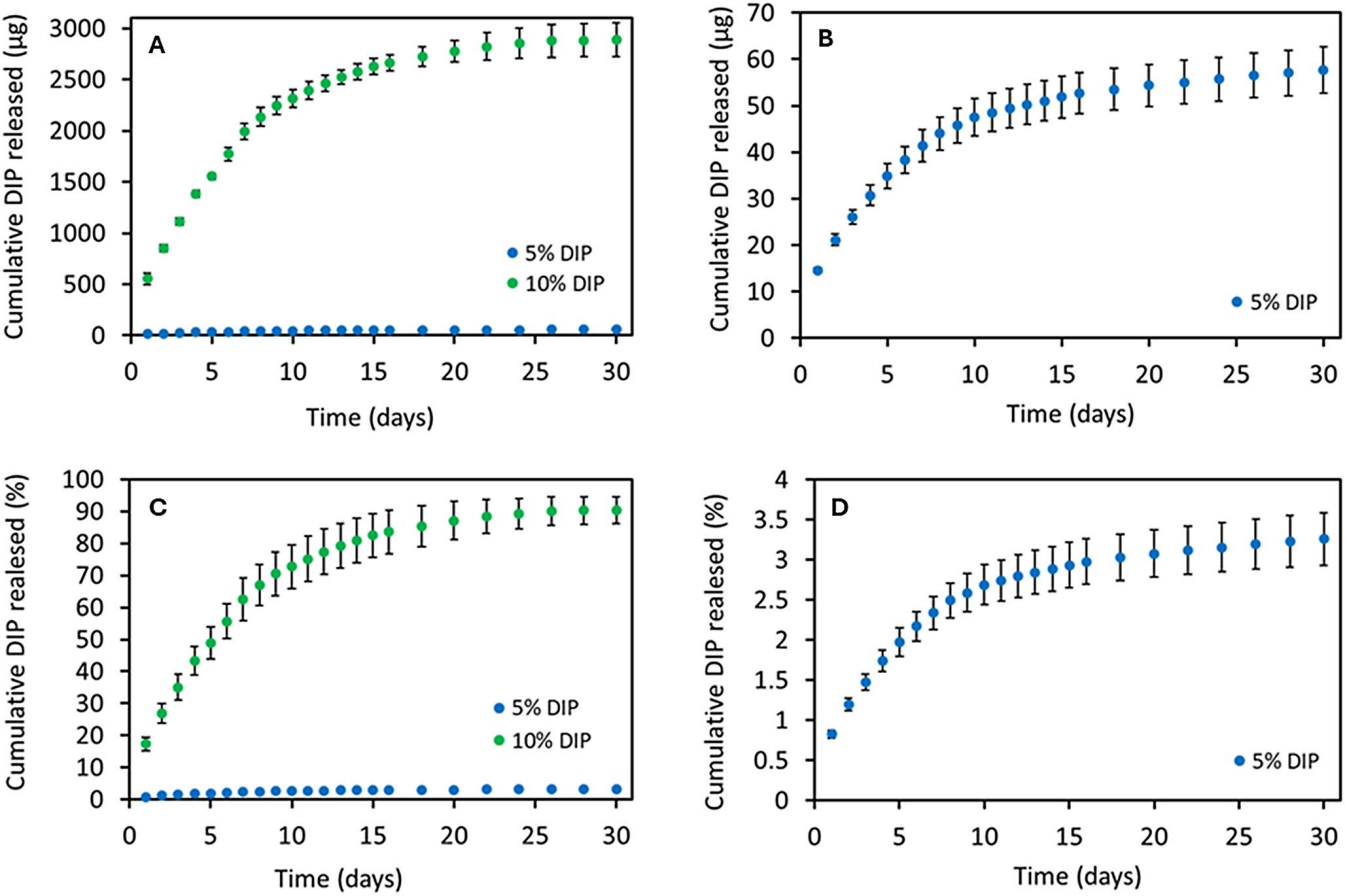
The profiles of cumulative in vitro DIP release from DIP-loaded 3D-printed scaffolds up to 30 days in PBS at 37 °C expressed in µg as function of time (**A**) and expressed in percentage as a function of initial DIP drug loading (**B**) (means ± SD, *n* = 4). Figures (**C** and **D**) represent the in vitro release profile of the 5% DIP-loaded 3D-printed meshes in a different scale expressed in µg as function of time and expressed in percentage as a function of initial DIP drug loading, respectively

In vitro release study was carried out for two scaffolds formulations containing 5% DIP and 10% DIP. DIP release from these 3D-printed scaffolds was evaluated for up to 30 days. Cumulative percentage release and cumulative release were calculated and presented in Fig. 8. Both formulations showed a slow drug release over 30 days without any obvious burst release. It is interesting to highlight that the scaffolds containing 10% DIP showed a higher DIP release (2888 µg) than samples containing 5% DIP (58 µg) (*p <* 0.0001). Similarly, in terms of percentage of release, the scaffolds containing 10% DIP released up to 90%, while the meshes containing 5% DIP were able to release only ca. 3%.

Monolithic-type implants typically show higher dissolution rates during the initial days of drug release experiments due to the dissolution and diffusion of the drug present on the implants’ surface. As the surface drug dissolves, it forms pores that allow the release medium to penetrate deeper into the matrix, triggering the release of drug embedded in the inner layers of the structure. Consequently, implants with lower drug loadings exhibit slower release kinetics. This behaviour, previously reported in the literature for TPU combined with other hydrophilic and hydrophobic drugs [26, 27, 70], may also explain the significant difference observed in this work between scaffolds containing 5% DIP (lower drug loading) and 10% DIP (higher drug loading). Additionally, this effect is even more pronounced with DIP due to its strong hydrophobicity.

The results suggest that the materials can provide drug release for periods up to 30 days. This timeframe is ideal as it has been reported that medical implants as osseointegration normally takes place 30 days post-implantation [83–85].

### In vitro biocompatibility and osteogenesis studies

**Fig. 9:**
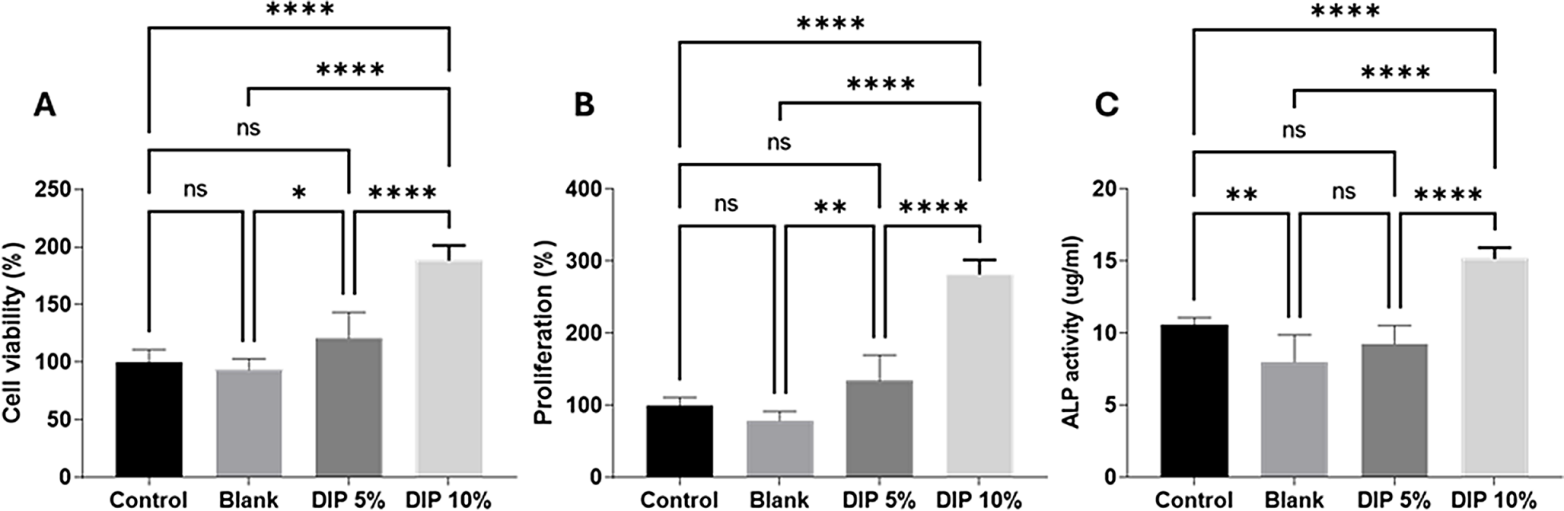
MC3T3-E1 cell biocompatibility and osteogenesis when cultured on tissue culture plastic (control), blank TPU (blank), or TPU containing 5% (DIP 5%) or 10% (DIP 10%) DIP. (**A**) Cell viability as measured by MTT assay and expressed as a percentage of control cells. (**B**) cell proliferation as measured by PicoGreen assay of total DNA content and expressed as a percentage of control cells. (**C**) osteogenic differentiation as measured by ALP activity

The effect of the 3D-printed samples on the biocompatibility and osteogenesis of MC3T3-E1 cells was evaluated. Cell viability was assessed using an MTT assay (Fig. 9a) which showed that viability was highest on the TPU DIP 10% discs (*p* < 0.0001 compared to both control cells and blank scaffold). There was a trend towards higher viability on the TUP DIP 5% but this was not statistically significant. Similarly, proliferation of MC3T3-E1 cells as indicated by the amount of dsDNA on each materials was significantly higher on TPU DIP 10% compared with control cells and blank material (both *p* < 0.0001) and non-significant increases in DNA were seen on TPU DIP 5% with respect to the control cells. However, the increase in DNA on the DIP 5% scaffold was statistically significantly larger than on blank material (*p* = 0.0015) To evaluate osteogenesis, the ALP activity of each sample was quantified. The ALP activity drastically increased in cells cultured on TPU DIP 10% formulation compared with control cells, blank and TPU DIP 5% as shown the Fig. 9C, suggesting that cells in high dipyridamole formulation were more proliferative as compared to the other experimental groups.

The results of this study demonstrate that native TPU can support osteoblast proliferation and differentiation at levels similar to tissue culture plastic without any detrimental effect on the cells. Previous studies have also shown this, confirming that TPU alone supported proliferation and ALP activity in human mesenchymal stem cells [86] although most studies seek to add an angiogenic or osteogenic bioactive such as nano-hydroxyapatite or rhBMP-2 [86–88]. In the current study, DIP was investigated as a way of enhancing osteogenesis and results showed there was a statistically significant increase in cell proliferation at both 5% and 10% DIP over blank TPU and enhanced ALP activity at the higher concentration of DIP.

In the literature some studies revealed the benefit effect of DIP in osteoblast maintenance and proliferation [52, 54], which could be due to the described effect of dipyridamole which regulates bone metabolism by increasing extracellular adenosine levels which activate adenosine A2AR, which is important for bone regeneration [54]. Specifically, some studies suggested that dipyridamole diminishes bone resorption by an adenosine receptor-mediated mechanism [52, 54]. Additionally, DIP was previously loaded into 3D-printed scaffolds for bone regeneration and evaluated in vivo [53]. However, in this experiment, 3D-printed scaffolds were coated with a DIP formulation. This approach requires an additional step, and the coating does not provide sustained drug release. Despite these limitations, this work demonstrated the efficacy of incorporating DIP into biomaterials for bone regeneration [53]. The implants were placed in calvarial bone in a sheep model, showing that after 3- and 6-weeks post-implantation, the drug provided increased bone formation across the implants. In this work the implants provided improved healing in the implant margins as well as within the outer and inner core of the implant. In this work it was hypothesized that DIP promote cell differentiation while enhancing pro-osteogenic influences [53].

The excellent cell proliferation might be also attributed to the hydrophilicity of the formulations as a main characteristic of biomaterials has a significant impact on cell attachment and cell growth. Therefore, the improved hydrophilicity of TPU DIP 10% [38] is also a key reason for higher cell viability and high osteogenesis. The present study describes a preliminary study on the feasibility of combining TPU with DIP via FDM to produce implants for bone-tissue engineering. More work is required to evaluate how these materials can be integrated with bone tissue in vivo as the resulting devices were only tested in cells. However, these promising results suggest that TPU/DIP implants could improve bone regeneration. This combined with the versatility of 3D-printing opens the possibility of preparing implants adapted to patient’s need at the point of care.

## Data Availability

The datasets generated during and/or analyzed during the current study are available from the corresponding author on reasonable request.
